# 4-Carbamoylpiperidinium 2-carb­oxy­benzoate–benzene-1,2-dicarb­oxy­lic acid (1/1)

**DOI:** 10.1107/S1600536811003825

**Published:** 2011-02-05

**Authors:** Graham Smith, Urs D. Wermuth

**Affiliations:** a Faculty of Science and Technology, Queensland University of Technology, GPO Box 2434, Brisbane, Queensland 4001, Australia; b School of Biomolecular and Physical Sciences, Griffith University, Nathan, Queensland 4111, Australia

## Abstract

The asymmetric unit of the title salt adduct, C_6_H_13_N_2_O^+^·C_8_H_5_O_4_^−^·C_8_H_6_O_4_, comprises one isonipecotamide cation, a hydrogen phthalate anion and a phthalic acid adduct mol­ecule. These form a two-dimensional hydrogen-bonded network through head-to-tail cation–anion–adduct mol­ecule inter­actions which include a cyclic heteromolecular amide–carboxyl­ate motif [graph set *R*_2_^2^(8)], conjoint cyclic *R*_2_^2^(6) and *R*_3_^3^(10) piperidinium N—H⋯O_carbox­yl_ associations, as well as strong carboxyl O—H⋯O_carbox­yl_ hydrogen bonds.

## Related literature

For structural data on isonipecotamide salts, see: Smith *et al.* (2010 ▶); Smith & Wermuth (2010*a* ▶,*b* ▶,*c* ▶,*d* ▶, 2011 ▶). For the crystal structure of *o*-phthalic acid, see: Ermer (1981 ▶). For hydrogen-bonding graph-set analysis, see: Etter *et al.* (1990 ▶).
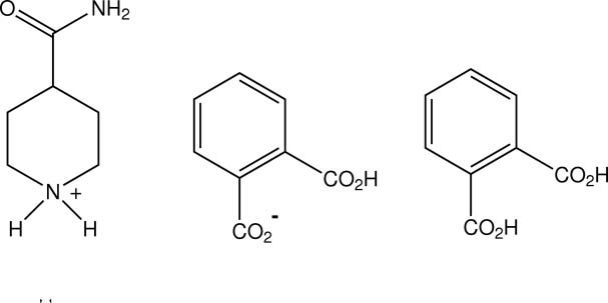


## Experimental

### 

#### Crystal data

C_6_H_13_N_2_O^+^·C_8_H_5_O_4_^−^·C_8_H_6_O_4_
*M* *_r_* = 460.43
Triclinic, 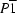

*a* = 8.7857 (4) Å
*b* = 11.7907 (6) Å
*c* = 12.3188 (6) Å
α = 62.496 (5)°
β = 85.916 (4)°
γ = 82.604 (4)°
*V* = 1122.36 (11) Å^3^
*Z* = 2
Mo *K*α radiation
μ = 0.11 mm^−1^
*T* = 200 K
0.40 × 0.30 × 0.18 mm

#### Data collection

Oxford Diffraction Gemini-S CCD-detector diffractometer
Absorption correction: multi-scan (*CrysAlis PRO*; Oxford Diffraction, 2010 ▶) *T*_min_ = 0.923, *T*_max_ = 0.980
13586 measured reflections
4401 independent reflections
3444 reflections with *I* > 2σ(*I*)
*R*_int_ = 0.024

#### Refinement

*R*[*F* ^2^ > 2σ(*F* ^2^)] = 0.037
*wR*(*F* ^2^) = 0.094
*S* = 1.07
4401 reflections
326 parameters
H atoms treated by a mixture of independent and constrained refinement
Δρ_max_ = 0.25 e Å^−3^
Δρ_min_ = −0.22 e Å^−3^

### 

Data collection: *CrysAlis PRO* (Oxford Diffraction, 2010 ▶); cell refinement: *CrysAlis PRO*; data reduction: *CrysAlis PRO*; program(s) used to solve structure: *SHELXS97* (Sheldrick, 2008 ▶); program(s) used to refine structure: *SHELXL97* (Sheldrick, 2008 ▶) within *WinGX* (Farrugia, 1999 ▶); molecular graphics: *PLATON* (Spek, 2009 ▶); software used to prepare material for publication: *PLATON*.

## Supplementary Material

Crystal structure: contains datablocks global, I. DOI: 10.1107/S1600536811003825/wn2419sup1.cif

Structure factors: contains datablocks I. DOI: 10.1107/S1600536811003825/wn2419Isup2.hkl

Additional supplementary materials:  crystallographic information; 3D view; checkCIF report

## Figures and Tables

**Table 1:** Hydrogen-bond geometry (Å, °)

*D*—H⋯*A*	*D*—H	H⋯*A*	*D*⋯*A*	*D*—H⋯*A*
N1*C*—H11*C*⋯O21*A*	0.932 (19)	1.911 (19)	2.8287 (18)	167.7 (16)
N1*C*—H12*C*⋯O12*A*^i^	0.953 (18)	2.077 (17)	2.8519 (16)	137.4 (14)
N1*C*—H12*C*⋯O12*B*^i^	0.953 (18)	2.204 (17)	2.9606 (16)	135.6 (14)
N41*C*—H41*C*⋯O22*B*^ii^	0.979 (19)	1.994 (19)	2.9494 (17)	164.5 (16)
N41*C*—H42*C*⋯O11*B*^iii^	0.930 (18)	2.120 (19)	3.0122 (17)	160.3 (16)
O11*A*—H11*A*⋯O12*B*	1.00 (2)	1.57 (2)	2.5635 (15)	173 (2)
O21*B*—H21*B*⋯O41*C*^iv^	0.99 (2)	1.58 (2)	2.5644 (14)	171 (2)
O22*A*—H22*A*⋯O11*B*^i^	0.90 (2)	1.65 (2)	2.5363 (17)	170.8 (18)

Symmetry codes: (i) 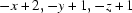; (ii) 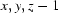; (iii) 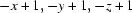; (iv) 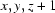.

**Fig. 1.**
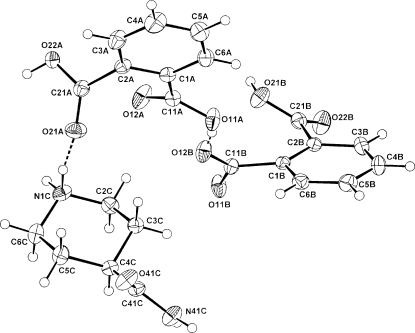
Molecular conformation for the INIPA cation (C), the hydrogen phthalate anion (B) and the phthalic acid adduct molecule (A) in the asymmetric unit. The inter-species hydrogen bonds are shown as dashed lines and displacement ellipsoids are drawn at the 40% probability level. Hydrogen atoms are shown as spheres of arbitrary radius.

**Fig. 2.**
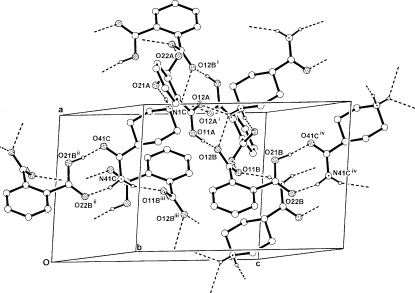
The hydrogen-bonded chain structure, showing the cyclic R22(8) amide–carboxyl and R22(6) piperidinium–carboxyl cation–anion associations. Non-associative H atoms have been omitted and hydrogen bonds are shown as dashed lines. For symmetry codes, see Table 1.

## supplementary crystallographic information

### Comment

The amide piperidine-4-carboxamide (isonipecotamide, INIPA) has provided the structures of proton-transfer compounds with a range of organic acids, mainly aromatic (Smith & Wermuth, 2010*a*,*b*,*c*,*d*, 2011; Smith *et al.*, 2010). The title compound, the salt adduct, C_6_H_13_N_2_O^+^ C_8_H_5_O_4_^-^ . C_8_H_6_O_4_, was obtained from the 1:1 stoichiometric reaction of phthalic acid with INIPA in methanol and the crystal structure is reported here; it represents the first example of a salt–adduct of INIPA.

The asymmetric unit (Fig. 1) comprises an isonipecotamide cation, (*C*), a hydrogen phthalate anion (*B*) and a phthalic acid adduct molecule (*A*), which together form a two-dimensional hydrogen-bonded network through head-to-tail cation–anion–adduct molecule interactions (Table 1). These include a cyclic heteromolecular amide–carboxylate motif [graph set R_2_^2^(8) (Etter *et al.*, 1990)], conjoint cyclic R_2_^2^(6) and R_3_^3^(10) piperidinium N—H···O_carboxyl_ associations, as well as strong carboxylic acid O—H···O_carboxyl_ hydrogen bonds (Fig. 2). There is no occurrence of the cyclic homomolecular amide–amide dimer motif association, such as is found in the INIPA salts of the 2-nitro-, 4-nitro- and 3,5-dinitrobenzoic acids (Smith & Wermuth, 2010*b*) or of biphenyl-4,4'-disulfonic acid (Smith *et al.*, 2010).

In the hydrogen phthalate anion (*B*) and the phthalic acid adduct molecule (*A*), the carboxyl substituent groups are rotated by differing degrees out of the planes of the benzene rings [torsion angles C1—C2—C21—O22 and C2—C1—C11—O11:  -147.67 (6) and 52.9 (2)° [for *B*)] and -117.75 (15) and -157.57 (14)° [for *A*)], which compare with 20.3 (1)° for the parent acid molecule which has two-fold rotational symmetry (Ermer, 1981).

### Experimental

The title compound was synthesized by heating together under reflux for 10 minutes, 1 mmol quantities of piperidine-4-carboxamide (isonipecotamide) and phthalic acid in 50 ml of methanol. After concentration to *ca* 30 ml, partial room temperature evaporation of the hot-filtered solution gave colourless plates of the title compound, from which a specimen was cleaved for the X-ray crystallographic analysis.

### Refinement

Hydrogen atoms involved in hydrogen-bonding interactions were located by difference methods and their positional and isotropic displacement parameters were refined. Other H-atoms were included in the refinement at calculated positions using a riding-model approximation [C—H = 0.93–0.98 Å] and with *U*_iso_(H) = 1.2*U*_eq_(C).

### Crystal data

**Table:**

C_6_H_13_N_2_O^+^·C_8_H_5_O_4_^−^·C_8_H_6_O_4_	*Z* = 2
*M**_r_* = 460.43	*F*(000) = 484
Triclinic, *P*1	*D*_x_ = 1.362 Mg m^−^^3^
Hall symbol: -P 1	Mo *K*α radiation, λ = 0.71073 Å
*a* = 8.7857 (4) Å	Cell parameters from 6939 reflections
*b* = 11.7907 (6) Å	θ = 3.2–28.7°
*c* = 12.3188 (6) Å	µ = 0.11 mm^−^^1^
α = 62.496 (5)°	*T* = 200 K
β = 85.916 (4)°	Plate, colourless
γ = 82.604 (4)°	0.40 × 0.30 × 0.18 mm
*V* = 1122.36 (11) Å^3^	

### Data collection

**Table:**

Oxford Diffraction Gemini-S CCD-detector diffractometer	4401 independent reflections
Radiation source: Enhance (Mo) X-ray source	3444 reflections with *I* > 2σ(*I*)
graphite	*R*_int_ = 0.024
Detector resolution: 16.077 pixels mm^-1^	θ_max_ = 26.0°, θ_min_ = 3.3°
ω scans	*h* = −10→10
Absorption correction: multi-scan (*CrysAlis PRO*; Oxford Diffraction, 2010)	*k* = −14→14
*T*_min_ = 0.923, *T*_max_ = 0.980	*l* = −15→15
13586 measured reflections	

### Refinement

**Table:**

Refinement on *F*^2^	Primary atom site location: structure-invariant direct methods
Least-squares matrix: full	Secondary atom site location: difference Fourier map
*R*[*F*^2^ > 2σ(*F*^2^)] = 0.037	Hydrogen site location: inferred from neighbouring sites
*wR*(*F*^2^) = 0.094	H atoms treated by a mixture of independent and constrained refinement
*S* = 1.07	*w* = 1/[σ^2^(*F*_o_^2^) + (0.0531*P*)^2^] where *P* = (*F*_o_^2^ + 2*F*_c_^2^)/3
4401 reflections	(Δ/σ)_max_ = 0.001
326 parameters	Δρ_max_ = 0.25 e Å^−^^3^
0 restraints	Δρ_min_ = −0.22 e Å^−^^3^

### Special details

**Table:**

Geometry. Bond distances, angles *etc*. have been calculated using the rounded fractional coordinates. All su's are estimated from the variances of the (full) variance-covariance matrix. The cell e.s.d.'s are taken into account in the estimation of distances, angles and torsion angles
Refinement. Refinement of *F*^2^ against ALL reflections. The weighted *R*-factor *wR* and goodness of fit *S* are based on *F*^2^, conventional *R*-factors *R* are based on *F*, with *F* set to zero for negative *F*^2^. The threshold expression of *F*^2^ > σ(*F*^2^) is used only for calculating *R*-factors(gt) *etc*. and is not relevant to the choice of reflections for refinement. *R*-factors based on *F*^2^ are statistically about twice as large as those based on *F*, and *R*- factors based on ALL data will be even larger.

### Fractional atomic coordinates and isotropic or equivalent isotropic displacement parameters (Å^2^)

**Table:**

	*x*	*y*	*z*	*U*_iso_*/*U*_eq_	
O41C	0.74423 (12)	0.61632 (11)	−0.02736 (9)	0.0416 (3)	
N1C	1.00683 (14)	0.42772 (13)	0.35653 (11)	0.0325 (4)	
N41C	0.52124 (15)	0.58014 (14)	0.08017 (12)	0.0383 (4)	
C2C	0.85240 (17)	0.48482 (14)	0.37702 (12)	0.0316 (5)	
C3C	0.77366 (16)	0.56671 (13)	0.25569 (12)	0.0279 (4)	
C4C	0.75894 (15)	0.48525 (13)	0.19015 (12)	0.0255 (4)	
C5C	0.91666 (17)	0.42485 (15)	0.17252 (13)	0.0352 (5)	
C6C	0.99655 (17)	0.34593 (15)	0.29489 (13)	0.0355 (5)	
C41C	0.67380 (16)	0.56532 (14)	0.07155 (12)	0.0287 (4)	
O11A	0.80799 (12)	0.85845 (10)	0.30180 (10)	0.0382 (3)	
O12A	1.01504 (12)	0.71996 (11)	0.38418 (9)	0.0457 (4)	
O21A	1.15402 (13)	0.64611 (10)	0.19609 (10)	0.0428 (4)	
O22A	1.34884 (12)	0.66738 (10)	0.28975 (10)	0.0350 (3)	
C1A	1.04746 (16)	0.90657 (13)	0.19787 (11)	0.0257 (4)	
C2A	1.18683 (15)	0.85461 (13)	0.16830 (12)	0.0273 (4)	
C3A	1.28238 (18)	0.93696 (15)	0.07974 (14)	0.0388 (5)	
C4A	1.2394 (2)	1.06857 (16)	0.02235 (15)	0.0456 (5)	
C5A	1.10150 (19)	1.11900 (15)	0.05043 (14)	0.0402 (5)	
C6A	1.00445 (17)	1.03847 (13)	0.13774 (12)	0.0321 (4)	
C11A	0.95520 (16)	0.81938 (13)	0.30320 (12)	0.0270 (4)	
C21A	1.22700 (16)	0.71181 (14)	0.22139 (12)	0.0285 (4)	
O11B	0.55460 (12)	0.56551 (9)	0.65124 (9)	0.0375 (3)	
O12B	0.70819 (11)	0.67951 (10)	0.49932 (9)	0.0365 (3)	
O21B	0.62518 (11)	0.72888 (11)	0.75928 (9)	0.0380 (3)	
O22B	0.39403 (12)	0.75761 (11)	0.83676 (9)	0.0443 (4)	
C1B	0.47554 (15)	0.78671 (12)	0.53468 (12)	0.0234 (4)	
C2B	0.41733 (15)	0.82601 (12)	0.62266 (12)	0.0250 (4)	
C3B	0.30225 (17)	0.92762 (14)	0.59082 (14)	0.0332 (5)	
C4B	0.24536 (18)	0.99166 (14)	0.47329 (15)	0.0400 (5)	
C5B	0.30399 (18)	0.95471 (14)	0.38598 (14)	0.0380 (5)	
C6B	0.41770 (17)	0.85304 (13)	0.41663 (13)	0.0309 (4)	
C11B	0.58924 (15)	0.66926 (13)	0.56461 (12)	0.0251 (4)	
C21B	0.47720 (16)	0.76662 (13)	0.75001 (12)	0.0282 (4)	
H4C	0.69780	0.41540	0.24300	0.0310*	
H11C	1.068 (2)	0.4933 (17)	0.3102 (16)	0.051 (5)*	
H12C	1.0542 (19)	0.3761 (16)	0.4339 (16)	0.048 (5)*	
H21C	0.86350	0.53720	0.41730	0.0380*	
H22C	0.79060	0.41670	0.42960	0.0380*	
H31C	0.83260	0.63760	0.20490	0.0330*	
H32C	0.67250	0.60230	0.26930	0.0330*	
H41C	0.461 (2)	0.6370 (17)	0.0071 (17)	0.058 (5)*	
H42C	0.475 (2)	0.5356 (17)	0.1562 (17)	0.054 (5)*	
H51C	0.90570	0.36990	0.13500	0.0420*	
H52C	0.97860	0.49200	0.11810	0.0420*	
H61C	0.93950	0.27400	0.34670	0.0430*	
H62C	1.09880	0.31190	0.28190	0.0430*	
H3A	1.37520	0.90370	0.05900	0.0470*	
H4A	1.30450	1.12320	−0.03570	0.0550*	
H5A	1.07320	1.20730	0.01080	0.0480*	
H6A	0.91060	1.07260	0.15610	0.0380*	
H11A	0.762 (2)	0.793 (2)	0.3786 (19)	0.078 (6)*	
H22A	1.374 (2)	0.583 (2)	0.3154 (18)	0.072 (6)*	
H3B	0.26280	0.95300	0.64920	0.0400*	
H4B	0.16780	1.05940	0.45310	0.0480*	
H5B	0.26690	0.99830	0.30660	0.0460*	
H6B	0.45630	0.82850	0.35750	0.0370*	
H21B	0.664 (2)	0.691 (2)	0.844 (2)	0.081 (7)*	

### Atomic displacement parameters (Å^2^)

**Table:**

	*U*^11^	*U*^22^	*U*^33^	*U*^12^	*U*^13^	*U*^23^
O41C	0.0306 (6)	0.0590 (7)	0.0233 (5)	−0.0098 (5)	0.0002 (4)	−0.0077 (5)
N1C	0.0310 (7)	0.0316 (7)	0.0255 (6)	−0.0079 (6)	−0.0069 (6)	−0.0032 (6)
N41C	0.0285 (7)	0.0545 (9)	0.0257 (7)	−0.0019 (6)	−0.0009 (6)	−0.0138 (7)
C2C	0.0382 (9)	0.0315 (8)	0.0255 (7)	−0.0033 (7)	−0.0024 (6)	−0.0133 (6)
C3C	0.0290 (8)	0.0272 (7)	0.0255 (7)	−0.0033 (6)	−0.0021 (6)	−0.0101 (6)
C4C	0.0256 (7)	0.0278 (7)	0.0216 (7)	−0.0058 (6)	0.0004 (5)	−0.0093 (6)
C5C	0.0340 (8)	0.0430 (9)	0.0282 (8)	0.0018 (7)	−0.0001 (6)	−0.0175 (7)
C6C	0.0301 (8)	0.0360 (9)	0.0356 (8)	0.0038 (7)	−0.0007 (6)	−0.0141 (7)
C41C	0.0280 (8)	0.0351 (8)	0.0256 (7)	−0.0061 (6)	−0.0006 (6)	−0.0155 (6)
O11A	0.0307 (6)	0.0306 (6)	0.0376 (6)	0.0049 (5)	0.0092 (5)	−0.0057 (5)
O12A	0.0312 (6)	0.0454 (7)	0.0307 (6)	0.0011 (5)	0.0005 (5)	0.0063 (5)
O21A	0.0453 (7)	0.0335 (6)	0.0459 (6)	−0.0133 (5)	−0.0085 (5)	−0.0116 (5)
O22A	0.0294 (6)	0.0271 (6)	0.0485 (6)	0.0029 (5)	−0.0075 (5)	−0.0178 (5)
C1A	0.0279 (7)	0.0259 (7)	0.0211 (7)	−0.0046 (6)	−0.0022 (6)	−0.0083 (6)
C2A	0.0253 (7)	0.0283 (8)	0.0249 (7)	−0.0062 (6)	−0.0011 (6)	−0.0083 (6)
C3A	0.0290 (8)	0.0378 (9)	0.0386 (8)	−0.0059 (7)	0.0051 (7)	−0.0083 (7)
C4A	0.0402 (10)	0.0367 (9)	0.0403 (9)	−0.0137 (8)	0.0060 (7)	0.0005 (8)
C5A	0.0446 (10)	0.0242 (8)	0.0376 (8)	−0.0041 (7)	−0.0045 (7)	−0.0016 (7)
C6A	0.0342 (8)	0.0277 (8)	0.0289 (7)	0.0005 (7)	−0.0034 (6)	−0.0089 (6)
C11A	0.0287 (8)	0.0271 (8)	0.0237 (7)	−0.0024 (6)	−0.0004 (6)	−0.0106 (6)
C21A	0.0242 (7)	0.0302 (8)	0.0282 (7)	−0.0058 (6)	0.0049 (6)	−0.0110 (6)
O11B	0.0377 (6)	0.0231 (5)	0.0381 (6)	0.0013 (5)	0.0143 (5)	−0.0056 (5)
O12B	0.0278 (6)	0.0373 (6)	0.0324 (5)	0.0012 (5)	0.0100 (4)	−0.0083 (5)
O21B	0.0281 (6)	0.0522 (7)	0.0244 (5)	−0.0004 (5)	−0.0019 (4)	−0.0104 (5)
O22B	0.0407 (7)	0.0563 (8)	0.0295 (6)	0.0060 (6)	0.0030 (5)	−0.0175 (5)
C1B	0.0209 (7)	0.0206 (7)	0.0265 (7)	−0.0045 (6)	0.0001 (5)	−0.0085 (6)
C2B	0.0244 (7)	0.0214 (7)	0.0281 (7)	−0.0050 (6)	0.0007 (6)	−0.0099 (6)
C3B	0.0347 (8)	0.0274 (8)	0.0377 (8)	0.0014 (7)	0.0010 (7)	−0.0165 (7)
C4B	0.0372 (9)	0.0265 (8)	0.0493 (10)	0.0096 (7)	−0.0118 (7)	−0.0132 (7)
C5B	0.0420 (9)	0.0306 (8)	0.0355 (8)	0.0016 (7)	−0.0176 (7)	−0.0092 (7)
C6B	0.0345 (8)	0.0299 (8)	0.0292 (7)	−0.0024 (7)	−0.0057 (6)	−0.0139 (6)
C11B	0.0238 (7)	0.0266 (7)	0.0232 (7)	−0.0022 (6)	0.0017 (6)	−0.0103 (6)
C21B	0.0308 (8)	0.0245 (7)	0.0273 (7)	−0.0033 (6)	0.0017 (6)	−0.0103 (6)

### Geometric parameters (Å, °)

**Table:**

O41C—C41C	1.2413 (17)	C5C—H51C	0.9700
O11A—C11A	1.3144 (18)	C5C—H52C	0.9700
O12A—C11A	1.2172 (19)	C6C—H61C	0.9700
O21A—C21A	1.220 (2)	C6C—H62C	0.9700
O22A—C21A	1.3062 (18)	C1A—C11A	1.4934 (19)
O11A—H11A	1.00 (2)	C1A—C2A	1.399 (2)
O22A—H22A	0.90 (2)	C1A—C6A	1.391 (2)
O11B—C11B	1.2537 (18)	C2A—C21A	1.500 (2)
O12B—C11B	1.2557 (17)	C2A—C3A	1.392 (2)
O21B—C21B	1.3140 (18)	C3A—C4A	1.387 (3)
O22B—C21B	1.2211 (17)	C4A—C5A	1.373 (3)
O21B—H21B	0.99 (2)	C5A—C6A	1.386 (2)
N1C—C6C	1.491 (2)	C3A—H3A	0.9300
N1C—C2C	1.494 (2)	C4A—H4A	0.9300
N41C—C41C	1.332 (2)	C5A—H5A	0.9300
N1C—H12C	0.953 (18)	C6A—H6A	0.9300
N1C—H11C	0.932 (19)	C1B—C2B	1.405 (2)
N41C—H42C	0.930 (18)	C1B—C11B	1.509 (2)
N41C—H41C	0.979 (19)	C1B—C6B	1.3918 (19)
C2C—C3C	1.5123 (19)	C2B—C3B	1.386 (2)
C3C—C4C	1.534 (2)	C2B—C21B	1.4954 (19)
C4C—C5C	1.523 (2)	C3B—C4B	1.383 (2)
C4C—C41C	1.5117 (19)	C4B—C5B	1.381 (2)
C5C—C6C	1.523 (2)	C5B—C6B	1.380 (2)
C2C—H21C	0.9700	C3B—H3B	0.9300
C2C—H22C	0.9700	C4B—H4B	0.9300
C3C—H31C	0.9700	C5B—H5B	0.9300
C3C—H32C	0.9700	C6B—H6B	0.9300
C4C—H4C	0.9800		
			
C11A—O11A—H11A	106.6 (12)	C2A—C1A—C11A	118.40 (13)
C21A—O22A—H22A	112.4 (12)	C3A—C2A—C21A	119.76 (14)
C21B—O21B—H21B	113.9 (11)	C1A—C2A—C3A	119.05 (15)
C2C—N1C—C6C	112.01 (12)	C1A—C2A—C21A	120.92 (12)
H11C—N1C—H12C	107.8 (15)	C2A—C3A—C4A	120.14 (16)
C6C—N1C—H11C	110.2 (12)	C3A—C4A—C5A	120.62 (16)
C2C—N1C—H11C	109.5 (12)	C4A—C5A—C6A	120.06 (17)
C2C—N1C—H12C	108.5 (11)	C1A—C6A—C5A	119.96 (15)
C6C—N1C—H12C	108.8 (13)	O12A—C11A—C1A	121.18 (13)
H41C—N41C—H42C	121.8 (16)	O11A—C11A—O12A	123.45 (13)
C41C—N41C—H42C	118.5 (11)	O11A—C11A—C1A	115.38 (13)
C41C—N41C—H41C	119.7 (11)	O22A—C21A—C2A	114.51 (14)
N1C—C2C—C3C	109.73 (11)	O21A—C21A—C2A	121.23 (13)
C2C—C3C—C4C	110.03 (13)	O21A—C21A—O22A	124.16 (16)
C5C—C4C—C41C	113.04 (12)	C2A—C3A—H3A	120.00
C3C—C4C—C5C	110.14 (12)	C4A—C3A—H3A	120.00
C3C—C4C—C41C	110.15 (13)	C5A—C4A—H4A	120.00
C4C—C5C—C6C	110.52 (12)	C3A—C4A—H4A	120.00
N1C—C6C—C5C	110.09 (14)	C4A—C5A—H5A	120.00
O41C—C41C—C4C	120.98 (13)	C6A—C5A—H5A	120.00
N41C—C41C—C4C	116.38 (12)	C5A—C6A—H6A	120.00
O41C—C41C—N41C	122.62 (13)	C1A—C6A—H6A	120.00
N1C—C2C—H21C	110.00	C2B—C1B—C6B	118.78 (14)
H21C—C2C—H22C	108.00	C6B—C1B—C11B	118.11 (13)
C3C—C2C—H21C	110.00	C2B—C1B—C11B	122.94 (12)
C3C—C2C—H22C	110.00	C1B—C2B—C21B	123.27 (13)
N1C—C2C—H22C	110.00	C3B—C2B—C21B	117.17 (13)
C4C—C3C—H31C	110.00	C1B—C2B—C3B	119.52 (13)
C2C—C3C—H32C	110.00	C2B—C3B—C4B	120.84 (15)
H31C—C3C—H32C	108.00	C3B—C4B—C5B	119.83 (16)
C4C—C3C—H32C	110.00	C4B—C5B—C6B	119.96 (14)
C2C—C3C—H31C	110.00	C1B—C6B—C5B	121.06 (14)
C41C—C4C—H4C	108.00	O11B—C11B—C1B	116.96 (12)
C5C—C4C—H4C	108.00	O12B—C11B—C1B	118.86 (13)
C3C—C4C—H4C	108.00	O11B—C11B—O12B	124.11 (15)
C4C—C5C—H51C	110.00	O21B—C21B—C2B	114.59 (12)
C4C—C5C—H52C	110.00	O22B—C21B—C2B	121.76 (13)
C6C—C5C—H51C	110.00	O21B—C21B—O22B	123.64 (13)
C6C—C5C—H52C	110.00	C2B—C3B—H3B	120.00
H51C—C5C—H52C	108.00	C4B—C3B—H3B	120.00
C5C—C6C—H61C	110.00	C3B—C4B—H4B	120.00
H61C—C6C—H62C	108.00	C5B—C4B—H4B	120.00
C5C—C6C—H62C	110.00	C4B—C5B—H5B	120.00
N1C—C6C—H61C	110.00	C6B—C5B—H5B	120.00
N1C—C6C—H62C	110.00	C1B—C6B—H6B	119.00
C2A—C1A—C6A	120.16 (13)	C5B—C6B—H6B	119.00
C6A—C1A—C11A	121.15 (13)		
			
C6C—N1C—C2C—C3C	−59.45 (17)	C1A—C2A—C21A—O22A	−117.75 (15)
C2C—N1C—C6C—C5C	58.30 (15)	C3A—C2A—C21A—O21A	−108.28 (17)
N1C—C2C—C3C—C4C	58.11 (16)	C3A—C2A—C21A—O22A	68.28 (18)
C2C—C3C—C4C—C5C	−57.23 (15)	C2A—C3A—C4A—C5A	−1.1 (3)
C2C—C3C—C4C—C41C	177.41 (11)	C3A—C4A—C5A—C6A	0.7 (3)
C3C—C4C—C5C—C6C	56.17 (17)	C4A—C5A—C6A—C1A	0.7 (2)
C41C—C4C—C5C—C6C	179.86 (14)	C6B—C1B—C2B—C3B	1.3 (2)
C3C—C4C—C41C—O41C	97.27 (18)	C6B—C1B—C2B—C21B	−176.43 (14)
C3C—C4C—C41C—N41C	−81.09 (18)	C11B—C1B—C2B—C3B	−173.80 (14)
C5C—C4C—C41C—O41C	−26.4 (2)	C11B—C1B—C2B—C21B	8.5 (2)
C5C—C4C—C41C—N41C	155.23 (16)	C2B—C1B—C6B—C5B	−0.8 (2)
C4C—C5C—C6C—N1C	−56.27 (17)	C11B—C1B—C6B—C5B	174.56 (14)
C6A—C1A—C2A—C3A	1.1 (2)	C2B—C1B—C11B—O11B	52.9 (2)
C6A—C1A—C2A—C21A	−172.89 (13)	C2B—C1B—C11B—O12B	−130.04 (15)
C11A—C1A—C2A—C3A	−172.71 (14)	C6B—C1B—C11B—O11B	−122.25 (15)
C11A—C1A—C2A—C21A	13.3 (2)	C6B—C1B—C11B—O12B	54.86 (19)
C2A—C1A—C6A—C5A	−1.6 (2)	C1B—C2B—C3B—C4B	−0.8 (2)
C11A—C1A—C6A—C5A	172.11 (14)	C21B—C2B—C3B—C4B	177.07 (14)
C2A—C1A—C11A—O11A	−157.57 (14)	C1B—C2B—C21B—O21B	34.0 (2)
C2A—C1A—C11A—O12A	23.0 (2)	C1B—C2B—C21B—O22B	−147.67 (16)
C6A—C1A—C11A—O11A	28.7 (2)	C3B—C2B—C21B—O21B	−143.76 (15)
C6A—C1A—C11A—O12A	−150.80 (15)	C3B—C2B—C21B—O22B	34.6 (2)
C1A—C2A—C3A—C4A	0.2 (2)	C2B—C3B—C4B—C5B	−0.3 (2)
C21A—C2A—C3A—C4A	174.29 (15)	C3B—C4B—C5B—C6B	0.8 (3)
C1A—C2A—C21A—O21A	65.69 (19)	C4B—C5B—C6B—C1B	−0.3 (2)

### Hydrogen-bond geometry (Å, °)

**Table:**

*D*—H···*A*	*D*—H	H···*A*	*D*···*A*	*D*—H···*A*
N1C—H11C···O21A	0.932 (19)	1.911 (19)	2.8287 (18)	167.7 (16)
N1C—H12C···O12A^i^	0.953 (18)	2.077 (17)	2.8519 (16)	137.4 (14)
N1C—H12C···O12B^i^	0.953 (18)	2.204 (17)	2.9606 (16)	135.6 (14)
N41C—H41C···O22B^ii^	0.979 (19)	1.994 (19)	2.9494 (17)	164.5 (16)
N41C—H42C···O11B^iii^	0.930 (18)	2.120 (19)	3.0122 (17)	160.3 (16)
O11A—H11A···O12B	1.00 (2)	1.57 (2)	2.5635 (15)	173 (2)
O21B—H21B···O41C^iv^	0.99 (2)	1.58 (2)	2.5644 (14)	171 (2)
O22A—H22A···O11B^i^	0.90 (2)	1.65 (2)	2.5363 (17)	170.8 (18)
C3B—H3B···O11A^v^	0.93	2.55	3.365 (2)	146
C4C—H4C···O11B^iii^	0.98	2.53	3.2159 (17)	127
C2C—H21C···O12A	0.97	2.54	3.317 (2)	137
C2C—H21C···O12B	0.97	2.55	3.364 (2)	142
C6C—H62C···O21B^i^	0.97	2.47	3.4265 (19)	168

Symmetry codes: (i) −*x*+2, −*y*+1, −*z*+1; (ii) *x*, *y*, *z*−1; (iii) −*x*+1, −*y*+1, −*z*+1; (iv) *x*, *y*, *z*+1; (v) −*x*+1, −*y*+2, −*z*+1.
